# Effects of blocking of angiotensin system on the prevalence of metabolic syndrome in type 2 diabetic patients

**DOI:** 10.12669/pjms.291.2782

**Published:** 2013

**Authors:** Isam Hamo Mahmood, Mohammed Najim Abed, Marwan Mohammed Merkhan

**Affiliations:** 1 Isam Hamo Mahmood, PhD, Head of Pharmacology Department, Department of Pharmacology, College of Pharmacy, University of Mosul, Mosul, Iraq.; 2 Mohammed Najim Abed, M.Sc, Lecturer, Department of Pharmacology, College of Pharmacy, University of Mosul, Mosul, Iraq.; 3 Marwan Mohammed Merkhan, M.Sc, Lecturer, Department of Pharmacology, College of Pharmacy, University of Mosul, Mosul, Iraq.

**Keywords:** Type 2 diabetes mellitus, Metabolic Syndrome, Renin Angiotensin System, ACE-inhibitors, ARBs

## Abstract

***Objective:*** To evaluate prevalence of metabolic syndrome in hypertensive type 2 diabetic patients treated with antihypertensive drugs that inhibit renin angiotensin system.

***Methodology:*** Two groups of patients were included in this study. The first group involved 130 hypertensive type 2 diabetic patients taking enalapril, captopril (Converting Enzyme inhibitors), valsartan or telmisartan (Angiotensin II receptor blockers) as monotherapy whereas group 2 involved 92 type 2 diabetic patients with normal blood pressure. Metabolic syndrome was diagnosed according to criteria made by the US National Cholesterol Education Program Adult Treatment Panel III. Serum glucose concentration, serum triglycerides and HDL-cholesterol were measured by using special kits.

***Results:*** The percentage of patients having metabolic syndrome was lower in group 1 (58.47%) as compared with group 2 (73%). Waist circumferences, triglycerides and FBS were significantly lower in group 1 as compared with group 2. BP and HDL-cholesterol were significantly higher in group 1 as compared with group 2.

***Conclusion:*** Inhibition of RAS by converting enzyme inhibitors or angiotensin II receptor blockers captopril, enalapril, valsartan or telmisartan produce beneficial effects on the markers of metabolic syndrome and can reduce the frequency of metabolic syndrome in type 2 diabetic patients.

## Introduction

 The metabolic syndrome is clinically characterized by several inter-related clinical presentation like obesity, dyslipidemia, high BP, insulin resistance, impaired glucose tolerance or diabetes. Each of these is a risk factor of cardiovascular disease and diabetes, but combinations of them greatly increase the risk of cardiovascular diseases.^1^ Metabolic syndrome is common and is increasing in prevalence worldwide, largely attributed to increasing obesity and sedentary lifestyles, and now is both a public health and clinical problem.^2^

 Metabolic syndrome can pave the way to both diabetes and heart disease, two of the common and important chronic diseases today.^3^^,^^4^ The risk of type 2 diabetes and heart diseases increased from 9-30 times over the normal population for diabetes and from 2-4 times that of the normal population for heart disease.^5^ Traditional risk factors for diabetes include obesity, dyslipidemia, hyperglycemia and hypertension. The clustering of these metabolic abnormalities has long been recognized and is now commonly termed metabolic syndrome.^6^

 A strong association exist between hypertension and diabetes and the prevalence of diabetes and hypertension were positively associated with BMI.^7^^,^^8^ Insulin resistance appears to be central features of metabolic syndrome, which is associated with hypertension, hyperlipidemia, hypercoagulability, inflammation and ultimately atherosclerosis and cardiovascular disease.^9^ Both hyperglycemia and insulin activate the renin angiotensin system (RAS) by increasing the expression of angiotensinogen, angiotensin II, and the angiotensin II type 1 receptor, which may contribute to the development of hypertension in patients with insulin resistance.^10^

 RAS is involved not only in the etiology of hypertension but also in the development of obesity and insulin resistance, providing a potential causal link among these-co morbidities of the metabolic syndrome. In rodents, pharmacological or genetic disruption of RAS action prevents weight-gain, promotes insulin sensitivity and relieves hypertension, implying that the administration of angiotensin converting enzyme inhibitors (ACE-inhibitors) or angiotensin receptor blockers (ARBs) may present an effective treatment for the metabolic syndrome in humans.^11^

 The aim of the present study was to investigate the effect of inhibition of RAS by using four well known antihypertensive drugs that inhibit the activity of RAS on the frequency of metabolic syndrome in type 2 diabetic patients. 

## Methodology

 Two hundred and twenty two type 2 diabetic patients were included in this study. They were divided into 2 groups. The first group involved 130 hypertensive type 2 diabetic patients with a mean age of 56.17±9.42 years; whereas group 2 involved 92 normal blood pressure type 2 diabetic patients with mean age of 54.18±8.4 years. Group 1 patients were taking antihypertensive drugs enalapril, captopril (Converting Enzyme inhibitors), valsartan or telmisartan (Angiotensin II receptor blockers) as monotherapy. All patients were on therapy with antidiabetic agents glibenclamide or metformin or a combination of both drugs.

 The study was a case, comparative trial, performed in Al-Wafaa Diabetic Center in Mosul city during the period between June 1st, 2011 to June 1st, 2012. The study protocol was approved by scientific committees of Department of Pharmacology in Pharmacy College and Mosul Health Administration.

 Only type 2 diabetic patients with or without hypertension were included in this study. All type 1 diabetics were excluded. Patients with renal failure, Cushing syndrome or with ascites due to any reason were excluded. Patients with secondary hypertension, hepatic disease and hypothyroidism were also excluded after thorough clinical evaluation. Patients taking drugs other than enalapril, captopril, valsartan or telmisartan that may affect the results of the study such as hypolipidemic agents or corticosteroids were also excluded.

 Metabolic syndrome was diagnosed according to the US National Cholesterol Education Program Adult Treatment Panel III criteria^6^ which require at least three of the following criteria: Waist circumference > 102 cm (male), >88 cm (female). Triglyceride ≥ 1.7 mmol/l, HDL-cholesterol < 1.0 mmol/l (male), < 1.3 mmol/l (female), BP ≥ 130/ ≥85 mmHg. Fasting blood glucose ≥ 6.1 mmol/l.

 Waist circumference in cm was determined with a standard tape measure, as the point midway between the costal margin and iliac crest in the mid-axillary line, with the subject standing and breathing normally. Blood pressure was measured by sphygmomanometer; measurement was performed after at least five minutes of rest. Fasting serum glucose concentration was estimated by glucose oxidase peroxidase colorimetric method by using a kit supplied by Biocon (Germany). Serum triglycerides and HDL-cholesterol were measured by using special kits supplied by Biolabo (France).

***Statistical Methods:*** Data were expressed as mean ± SD and %. Unpaired t-test was used to compare data between group 1 and group 2. Values ≤ 0.05 were considered significant.

## Results

 Patient’s characteristics are showed in Table-I. The two groups were matched regarding age. BMI was significantly higher in group 2. The number and percentage of patients having metabolic syndrome in each group is shown in Table-II. The percentage of patients having metabolic syndrome was lower in group 1 (58.47%) as compared with group 2 (73%).

**Table-I:** Patients Characteristics

*Parameter*	*Group 1(130)*	*Group 2(92)*	*P value*
Age (y)	56.17±9.42	54.18±8.4	0.101
BMI (kg/ m^2^)	28.96±3.o2	31.26±4.55	0.000
Sex M F	62 47.7% 68 52.3%	41 44.56% 51 55.44%	
Duration of treatment of hypertension (Y)	4.0±1.83		

**Table-II:** Number of patients with metabolic syndrome in the 2 groups

*Parameter*	*Group 1 (130)*	*Group 2 (92)*
Metabolic Syndrome	76 58.47%	68 73%
Non Metabolic Syndrome	54 41.53%	24 27%

 Comparison between parameters of group 1 and group 2 appears in Table-III. Waist circumferences, triglycerides and FBS were significantly lower in group 1 as compared with group 2. BP and HDL-cholesterol were significantly higher in group 1 as compared with group 2.

## Discussion

 The results of the present study showed that the prevalence of metabolic syndrome (MS) in group 1 which involved diabetic patients treated with drugs that reduce RAS activity like enalapril, captopril, valsartan or telmisartan as monotherapy, was 58.47% which was less than figure of 73% obtained from group 2 which involved diabetic patients not treated with antihypertensive agents. This may indicate that these drugs have beneficial effects on the markers of MS.

 Many studies have demonstrated over activity of RAS in patients with metabolic syndrome, which are implicated in the occurrence of this syndrome.^11^^-^^13^ Studies support the presence of a local RAS in the adipose tissue, which may have an important role in the physiological regulation of this tissue and may have also a role in the pathophysiology of obesity and of obesity-related hypertension.^14^ Weisinger et al.^15^ reported that the RAS is functional within adipose tissue and angiotensin II, the active component of RAS, has been implicated in adipose tissue hypertrophy and insulin resistance. In addition to the exacerbation of hypertension, the RAS has been implicated in the etiology of obesity and insulin resistance, providing perhaps a pivotal link among obesity, diabetes and hypertension, all symptoms of metabolic syndrome.^16^^-^^18^

**Table-III:** Comparison between parameters of group 1 and group 2

*Markers*	*Group 1 (130)*	*Group 2 (92)*	*P value*
Waist Circumferences (cm)	92.38±7.24	95.6±6.51	0.001
FBS (mmol/l)	7.94±1.89	8.75±3.02	0.026
Systolic BP (mmHg)	138.19±9.8	133.08±7.67	0.000
Diastolic BP(mmHg)	81.88±5.07	77.25±5.12	0.000
Triglycerides (mmol/l)	1.86±0.77	2.14±1.02	0.028
HDL Cholesterol (mmol/l)	1.21±0.28	1.11±0.36	0.026

 It was found that adipose tissue synthesizes and secretes the major components of RAS.^19^ There is also evidence for over activation of adipose tissue RAS in obesity in rodents,^20^ and for a positive correlation between adipose tissue angiotensinogen levels and BMI in humans.^21^ Moreover, circulating levels of angiotensinogen correlate with BMI and estimated total adipose tissue-derived angiotensinogen in humans,^22^ suggesting an endocrine role for adipose angiotensinogen. Conversely, plasma and adipose angiotensinogen levels are decreased following weight-loss.^23^ Moreover, animal models with targeted inactivation of RAS genes exhibit improved insulin sensitivity and are protected from high-fat diet-induced obesity and insulin resistance.^24^

 In the present study FBS of group 1 was statistically lowered than that of group 2. Many experimental and clinical trials demonstrated a reduction of obesity, insulin resistance, BP, diabetes mellitus (DM), and lipid components which are regarded as components of metabolic syndrome by using drugs that reduce the activity of RAS. Data from clinical trials suggest that RAS blockade not only reduces cardiovascular risk in patients with DM but may also prevent or delay DM onset in at-risk subjects and reducing progression of the disease.^25^ Further, RAS blockade also ameliorates insulin resistance and glucose intolerance in several rodent models of obesity.^26^ Angiotensin converting enzyme inhibition has been shown to improve insulin sensitivity and glycemic control in diabetic patients^27^ and to result in a 14% relative reduction in the incidence of new-onset type 2 diabetes.^28^ The mechanisms by which ACE inhibitors improve insulin sensitivity appear to be due in part to increased glucose uptake in skeletal muscle via enhanced synthesis and translocation of glucose transporter 4 protein to the cell surface, an effect that is promoted by up-regulation of tyrosine phosphorylation of IRS-1 and enhanced bradykinin and NO activities.^29^

 In the present study waist circumference and BMI values were lowered in group 1 as compared with those of group 2 suggesting contributing effects of RAS inhibition. RAS inhibition can reduce body weight,^30^^,^^31^ and abdominal obesity.^32^ In a study, performed by Weisinger et al.^15^ captopril, was used to study the development of diet-induced obesity and insulin resistance in obesity prone C57BL/6J mice, captopril treatment decreased body weight in the first 2 weeks of treatment. Food intake of captopril-treated mice was similar to control mice prior to weight loss and was decreased after weight loss. Glucose tolerance was improved in captopril-treated mice. Captopril-treated mice had less epididymal fat than control mice. Relative to control mice, mice administered captopril had a higher plasma concentration of adiponectin and lower concentrations of leptin and non-esterified fatty acids (NEFA). The results indicate that captopril both induced weight loss and improved insulin sensitivity.

 The results in the present study showed higher values of blood pressure in the group 1 as compared with group 2. This may be due to the fact that, the patients in group 1 were hypertensive and not all of them became normotensive by treatment with antihypertensive drugs.

 In the present study serum triglyceride was lowered and HDL-Cholesterol was higher in group 1 than that of group 2 indicating favorable effects of the antihypertensive agents on these 2 lipid parameters. Many studies have also demonstrated a beneficial effects of these agents on serum glucose concentration and lipid profile^33^^-^^37^ which were in agreement with the present study.

## Conclusions

 Inhibition of RAS by converting enzyme inhibitors or angiotensin II receptor blockers captopril, enalapril, valsartan or telmisartan produced beneficial effects on the markers of metabolic syndrome and can reduce the frequency of metabolic syndrome in type 2 diabetic patients.
